# Consolidating evidence on the effectiveness of interventions promoting fruit and vegetable consumption: an umbrella review

**DOI:** 10.1186/s12966-020-01046-y

**Published:** 2021-01-11

**Authors:** Luke Wolfenden, Courtney Barnes, Cassandra Lane, Sam McCrabb, Hannah M. Brown, Sarah Gerritsen, Simon Barquera, Lesly Samara Véjar, Ana Munguía, Sze Lin Yoong

**Affiliations:** 1 School of Medicine and Public Health, The University of Newcastle, Hunter New England Population Health, Locked Bag 10 Wallsend NSW, Newcastle, NSW 2287 Australia; 2 Corporación Actuemos, Santiago, Chile; 3 School of Population Health, University of Auckland, Auckland, New Zealand; 4 Centro de Investigación en Nutrición y Salud, Instituto Nacional de Salud Pública, Cuernavaca, Mexico; 5 Swinburne University of Technology, School of Health Sciences, Hawthorn, VIC Australia

**Keywords:** Nutrition policy, Recommended dietary allowances, Diet, Public health, Health promotion

## Abstract

**Background:**

The overarching objective was to examine the effectiveness of intervention strategies to promote fruit and vegetable consumption. To do this, systematic review evidence regarding the effects of intervention strategies was synthesized; organized, where appropriate, by the setting in which the strategies were implemented. Additionally, we sought to describe gaps in the review of evidence; that is, where evidence regarding the effectiveness of recommended policy actions had not been systematically synthesised.

**Methods:**

We undertook a systematic search of electronic databases and the grey literature to identify systematic reviews describing the effects of any intervention strategy targeting fruit and/or vegetable intake in children or adults of any age.

**Results:**

The effects of 32 intervention strategies were synthesised from the 19 included reviews. The strategies were mapped across all three broad domains of the NOURISHING framework (i.e. food environment, food system and behaviour change communication), but covered just 14 of the framework’s 65 sub-policy areas. There was evidence supporting the effectiveness of 19 of the 32 intervention strategies. The findings of the umbrella review suggest that intervention strategies implemented within schools, childcare services, homes, workplaces and primary care can be effective, as can eHealth strategies, mass media campaigns, household food production strategies and fiscal interventions.

**Conclusions:**

A range of effective strategy options are available for policy makers and practitioners interested in improving fruit and/or vegetable intake. However, the effects of many strategies – particularly those targeting agricultural production practices, the supply chain and the broader food system – have not been reported in systematic reviews. Primary studies assessing the effects of these strategies, and the inclusion of such studies in systematic reviews, are needed to better inform national and international efforts to improve public health nutrition.

**Trial registration:**

The review protocol was deposited in a publicly available Open Science framework prior to execution of the search strategy. https://osf.io/unj7x/.

**Supplementary Information:**

The online version contains supplementary material available at 10.1186/s12966-020-01046-y.

## Background

Low fruit and vegetable consumption are a modifiable risk factor that is contributing to the rising international burden of non-communicable diseases [1, 2]. In 2017, 3.9 million deaths worldwide were attributed to inadequate fruit and vegetable intake [3]. Adequate intake of fruits and vegetables reduces the risk of a variety of chronic health conditions including hypertension, coronary heart disease, stroke [4] and type 2 diabetes [5]. The health promoting properties of fruits and vegetables can be attributed to their concentrations of bioactive compounds, including vitamins, minerals, antioxidants and fibre [6]. The concentration of these compounds may differ between fruits and vegetables, with fruits typically containing more dietary sugars, and vegetables more protein and fibre [6]. Although certain types of fruits or vegetables may be particularly beneficial for particular health outcomes – for example, cruciferous vegetables may reduce the risk of a number of specific cancers [7–9] – intake of both fruits and vegetables is recommended to promote good health [10].

The World Health Organization (WHO) recommends a combined consumption of at least 400 g (g) of fruits and vegetables per day [3]. However, current global fruit and vegetable intakes fall short of the WHO recommendations. A systematic analysis of 266 country-specific nutrition surveys worldwide found that, in 2010, global fruit intake was 81.3 g/day, with only two countries having mean intakes of at least 300 g/day [11]. There is a similar situation with youth – Global School-Based Student Health Survey data from 2004 to 2013 found that less than 30% of adolescents from 49 low- and middle-income countries (LMIC) met WHO minimum recommended levels of intake for fruits and vegetables [12].

Improving population intakes of fruits and vegetables represents a considerable challenge. The determinants of intake are complex, and nest within a dynamic food system where there is interaction of factors such as food production, supply and affordability, access, food environments and individual behaviours [13]. Several of these determinants, specifically those that relate to supply, access and affordability, are particularly acute in LMIC [13]. A range of global plans have been developed to prompt action to increase fruit and vegetable intake, including the WHO Global Strategy on Diet, Physical Activity and Health and, more recently, the WHO Global Action Plan for the Prevention and Control of Non Communicable Diseases (NCDs) 2013–2020, and the United Nations (UN) Decade of Action on Nutrition 2016–2025. Similarly, the NOURISHING framework of the World Cancer Research Fund International (WCRF) has been established to guide national efforts to improve public health nutrition. The framework specifies a range of interventions across 10 key policy areas in three key domains: food environment (i.e. food labelling standards), food system (i.e. supply chain actions) and behavioural change communication (i.e. nutrition counselling) [14, 15]. The framework was developed following a meeting of international experts; it draws on previously developed frameworks and aligns with international policy options to improve public health nutrition. It is also intended to facilitate the reporting and monitoring of policy actions internationally.

To date, government action to improve fruit and vegetable consumption has been variable. The Global Nutrition Policy Review found that just 63% of 167 countries with national nutrition polices included goals, targets or indicators focused on improving fruit and vegetable intake; also, initiatives to promote fruit and vegetable consumption varied considerably by WHO region [16]. The report found that the proportion of countries with school-based fruit and vegetable schemes had decreased markedly over the past decade, but among the WHO regions it was highest in Europe (51%), and lowest in Africa (13%) and the Eastern Mediterranean (16%). A 2018 audit of the WCRF NOURISHING database, which catalogues government nutrition policies and actions globally, reported 168 polices specifically designed to improve fruit and vegetable intake [13]. However, these policies focused almost entirely on two of the 10 domains of the framework: offering healthy food and setting standards in institutions and settings, and informing people about food and nutrition through public awareness initiatives [13]. Such findings are similar to earlier systematic reviews of nutrition policies in LMIC, which have reported that most global nutrition activities to improve fruit and vegetable intake are focused on public education and demonstrations [17].

To maximise the potential impact of investment in initiatives to improve fruit and vegetable intake, it is recommended that evidence be used to influence decision-making in health policy and practice. Rigorous systematic reviews aim to identify, capture and consider all relevant evidence [18, 19]. As such, the use of such reviews is recommended as the basis of health policy and practice decisions [20]. There is an ever-increasing pool of primary studies investigating the effects of fruit and vegetable intervention strategies; in recent years, numerous systematic reviews have been undertaken to synthesise this evidence [21–26]. Managing the information presented in large numbers of systematic reviews can be challenging for decision-makers. Unlike conventional systematic reviews, which synthesise the findings of individual primary studies, overviews of systematic reviews (also known as ‘umbrella’ reviews) can help to overcome these challenges by assessing and consolidating the findings of systematic reviews and meta-analyses [27]. Umbrella reviews provide a means of rapidly and efficiently synthesising evidence from a broad body of research, [27, 28] and can assist decision-makers in choosing from different intervention strategies; thus, they are frequently used as the basis for health policy and guideline development [29–32]. Umbrella reviews are also useful to identify gaps where policy or practice is being recommended in the absence of evidence (or contrary to evidence), or where beneficial intervention strategies exist but are not being routinely delivered.

Given the importance of fruit and vegetable intake to human health, and the breadth of strategies suggested to improve intake, we conducted an umbrella review to inform a background paper for the 2020 Food and Agriculture Organization of the UN (FAO)/WHO International Workshop on Vegetables and Fruits for Food and Quality of Life. The workshop seeks to support public sector organisations to develop national policies, strategies, regulatory frameworks and other intervention strategies, to improve population fruit and vegetable intake. The overarching objective is to examine the effectiveness of intervention strategies to promote fruit and vegetable consumption. To do this, we synthesized systematic review evidence regarding the effects of intervention strategies, organized (where appropriate) by the setting in which they were implemented. We also sought to describe gaps in the review of evidence (i.e. areas where evidence regarding the effectiveness of recommended policy actions had not been systematically synthesised). In both synthesis of evidence and identification of gaps, we used the recommended policy areas identified in the WCRF NOURISHING framework.

## Methods

The review was guided by recommendations for the conduct of umbrella reviews from the Joanna Briggs Institute [33] and the Cochrane Handbook [34]. The review protocol was deposited in a publicly available Open Science framework prior to execution of the search strategy (https://osf.io/unj7x/) [35]. The findings of the review are reported based on suggestions in the protocol for Preferred Reporting Items for Overviews of Reviews (PRIOR) [36].

### Inclusion and exclusion criteria

We included systematic reviews that assessed the effectiveness of intervention strategies on a measure of fruit and vegetable intake. We included one systematic review per intervention strategy identified. Consistent with recommendations in the Cochrane Handbook, where multiple reviews report the effects of the same intervention strategy, we selected the most recent high-quality review for inclusion. This approach meant that the number of systematic reviews included could not exceed the number of strategies reported in this umbrella review. However, in some instances, a single review was included that reported on the effects of multiple intervention strategies.

#### Design

We included systematic reviews with or without meta-analysis that described the effectiveness of any intervention strategies on a measure of fruit and/or vegetable intake in children or adults. Initially, we included only reviews of controlled trials; however, following initial citation screening, we modified the criteria to include reviews of any prospective evaluation design (with or without a parallel control or comparison group). The aim of this modification was to provide greater opportunity for the inclusion of reviews reporting the effects of macro-level intervention strategies (e.g. natural experiments following fiscal policy change) that may not be amenable to group allocation. All citations were rescreened using this modified criterion to ensure consistency with the screening method.

We excluded reviews comprising solely qualitative research, because they do not provide quantitative estimates of the effects of intervention strategies. Reviews published before 2011 were also excluded (as per Joanna Briggs Institute guidance), because those published in the past 10 years represent the contemporary evidence base and will capture primary research conducted over the previous 30 or so years [27].

Also excluded were reviews of intervention strategies on population groups selected on the basis of pre-existing comorbidities (e.g. those with type 2 diabetes, hypertension or cancer); reviews focused on strategies that targeted the treatment or management of eating disorders (e.g. anorexia nervosa or bulimia) or other diseases; reviews of intervention strategies undertaken in laboratories or other simulated contexts (e.g. laboratory-based experiments of infant feeding practices); reviews in which intervention strategies were defined only by the population group targeted and not characterised by setting, delivery modality or content; and reviews examining intervention mechanisms (e.g. theory-based mechanisms of effect), or in which the effects of intervention components were synthesised at the level of individual behaviour change techniques.

#### Outcomes

The primary outcome for the umbrella review was fruit and/or vegetable intake (referred to here as ‘fruit and vegetable intake’ unless otherwise specified), assessed via self-report, observational, biochemical or other measures, or via objective measures of fruit and vegetable purchases (as an accepted proxy). Specifically, fruit and vegetable intake is measured in, for example, grams, portions or serves, assessed using measures such as food diaries, dietary recalls, food frequency questionnaires, observation or other approaches (e.g. measures of plate waste or photographs). Before undertaking the search, we also included objective measures of purchases of fruits and vegetables (e.g. those supplied by food outlets or supermarkets) as a proxy for dietary intake. Such outcomes are more likely to be used in evaluations of intervention strategies at policy or food system level, and represent a reliable surrogate measure of intake. To be included, systematic reviews must have synthesised the effects of strategy types on any measure of fruit and vegetable intake across the included reviews, in narrative or quantitative form (e.g. meta-analysis). We excluded reviews that included primary studies that reported on fruit and vegetable outcomes as part of other dietary measures (e.g. total diet quality), but did not explicitly synthesis across primary studies. We also excluded measures of behavioural intention, preference or liking for fruits or vegetables.

### Search strategy

A search of peer reviewed and grey literature was undertaken on 11 June 2020 in the following bibliographic databases: MEDLINE, Embase, CINAHL, The Cochrane Library, Scopus and Academic Search Ultimate. We combined terms for ‘intervention strategy’, ‘systematic reviews’ and ‘fruits and vegetables’, and limited the search to the dates 2011 to 11 June 2020. Search strategies developed in MEDLINE were adapted for other databases by an information specialist (see Additional File for the detailed search strategy). We also searched all publications listed in the WHO e-Library of Evidence for Nutrition Actions (eLENA), and the WCRF NOURISHING website for any additional systematic reviews on fruit and vegetable intake; conducted targeted Google Scholar searches; and searched for grey literature using the search engine ‘Google’. Finally, we sent compiled lists of reviews identified through database searches to experts in the field of nutrition and public health from FAO and WHO, to identify any additional potentially eligible reviews.

### Review selection and data extraction

A single reviewer (LW) initially screened all citations, and excluded all clearly ineligible citations based on title. The remaining titles and abstracts were screened in duplicate (CB, CL) for eligibility. The full texts of all potentially relevant reviews were obtained and assessed against the inclusion criteria in duplicate (CB, CL). Manuscripts published in non-English languages were translated using ‘Google Translate’ and, if manuscripts were eligible for inclusion, a native language reviewer was sought to assist with screening and extraction. Disagreement regarding the eligibility of a review was resolved by discussion and consensus, or by consultation with an additional reviewer (LW).

Data were extracted independently and in duplicate by review authors (CB, CL, LW). The data extracted included the following information recommended by the Joanna Briggs Institute for the conduct of umbrella reviews: [27] citation details, objectives of the included review, review eligibility criteria (e.g. population, intervention, comparison and outcome characteristics, as well as setting or context), number of databases (including grey literature) sourced and searched, date range of database searching, number of included primary studies, instrument used to appraise the quality of primary studies and the rating of their quality, fruit and vegetable outcomes and effects reported by intervention strategy type and comparison, and method of synthesis or analysis. Consistent with recommendations for umbrella reviews, [27] extraction and presentation of findings and results was limited to those presented by the included systematic reviews (i.e. primary studies were not re-analysed).

Where a review reported separate syntheses of the effects of different intervention strategies, we extracted information describing the effects of each synthesis. If two or more reviews reported the effects of the same intervention strategies, we included only the findings of the most recent high-quality review. To do this, we assessed all reviews with a search date within two years of the most recent review, and selected the review with the highest quality rating. This meant that, for each intervention strategy, we included and reported only the findings of syntheses in the most contemporary and rigorous reviews available, and thus reduced the risk of any potential bias from overlapping reviews and primary studies.

Where reviews examined the effects of the same intervention strategy but in mutually exclusive population groups – for example, children and adults – we reported the effects in both groups. However, where reviews were undertaken on a population group and a subset of a population group – for example, children and girls only – we included only the more inclusive sample (i.e. in this example, children rather than girls only).

### Assessment of methodological quality of included reviews

We assessed the methodological quality of included reviews independently and in duplicate (SMc, HB), using the critical appraisal tool developed by the Joanna Briggs Institute [27, 37]. Discrepancies between reviewer assessments were resolved via consensus. The checklist requires assessment of the review by authors against 11 methodological criteria, each scored as ‘yes’, ‘no’, ‘unsure’ or ‘not applicable’. Additional file 2 provides details on how the scoring system was applied and details of the specific checklist items. Consistent with previous umbrella reviews, [37] we defined reviews as ‘low quality’ where 33% or less of the criteria were met, ‘medium quality’ where 34–66% of the criteria were met, and ‘high quality’ where 67% or more of the criteria were met. Criteria deemed ‘not applicable’ were not included in the denominator in assessments of review quality [37].

### Data analysis

We used basic frequencies to describe the search results; described excluded and included reviews in line with the Preferred Reporting Items for Systematic Reviews and Meta-Analyses (PRISMA) guidance [38]; and narratively described the characteristics of included reviews. Quality assessments of reviews are presented in tabular form for each included review. We included reviews employing both quantitative and narrative syntheses.

To describe the effectiveness of the intervention strategies we reported, where available, the point estimates of effect, measures of variability, *p*-values and measures of heterogeneity in any included meta-analysis. Where the included reviews had narratively synthesised intervention strategies, we extracted and reported statements within the review that best summarised their overall effects. Summary statements were agreed on by two authors (CB, CL or LW). Where reviews used both a formal measure of risk of bias or assessment of the quality of primary studies (e.g. Cochrane Risk of Bias tool), [39] and an evidence grading system, such as Grading of Recommendations, Assessment, Development and Evaluation (GRADE), [40] we reported findings from the grading system. This was because the evidence grading systems consider both primary study quality or risk of bias as well as other attributes to describe the overall body of evidence, and are recommended for use in health decision-making [41].

We used the WCRF NOURISHING framework [42] to describe evidence gaps, by comparing policy areas recommended for action in the framework to the effects of interventions strategies synthesised in the included reviews. Specifically, two authors (CB, CL) mapped descriptions of the intervention strategies synthesised in the included systematic reviews to the framework, based on descriptors provided on the WCRF website [42]. The WCRF NOURISHING framework covers a comprehensive package of policies to promote healthy eating across three broad domains: food environment, food system and behaviour change communication. Governments are encouraged to implement initiatives across each of the 10 evidence-informed policy areas (and associated sub-policy areas). Although the framework has a focus on actions to improve healthy eating overall, it has previously been used to characterise fruit and vegetable initiatives globally, [13] and many of the proposed policy areas could conceivably affect intake. Where reviews synthesized the effects of intervention strategies that exclusively aligned with a discrete NOURISHING sub-policy area, we denoted them as being ‘directly mapped’ to that policy area in the framework. Where reviews assessed the effects of intervention strategies that included related components inclusive of but not restricted to a policy area, we denoted them as being ‘indirectly mapped’ to that policy area. We also listed reviews of intervention strategies that could not be mapped to any sub-policy area of the framework. Finally, we described, broadly, the effects of intervention strategies mapped to each area of the framework.

## Results

**Fig. 1:**
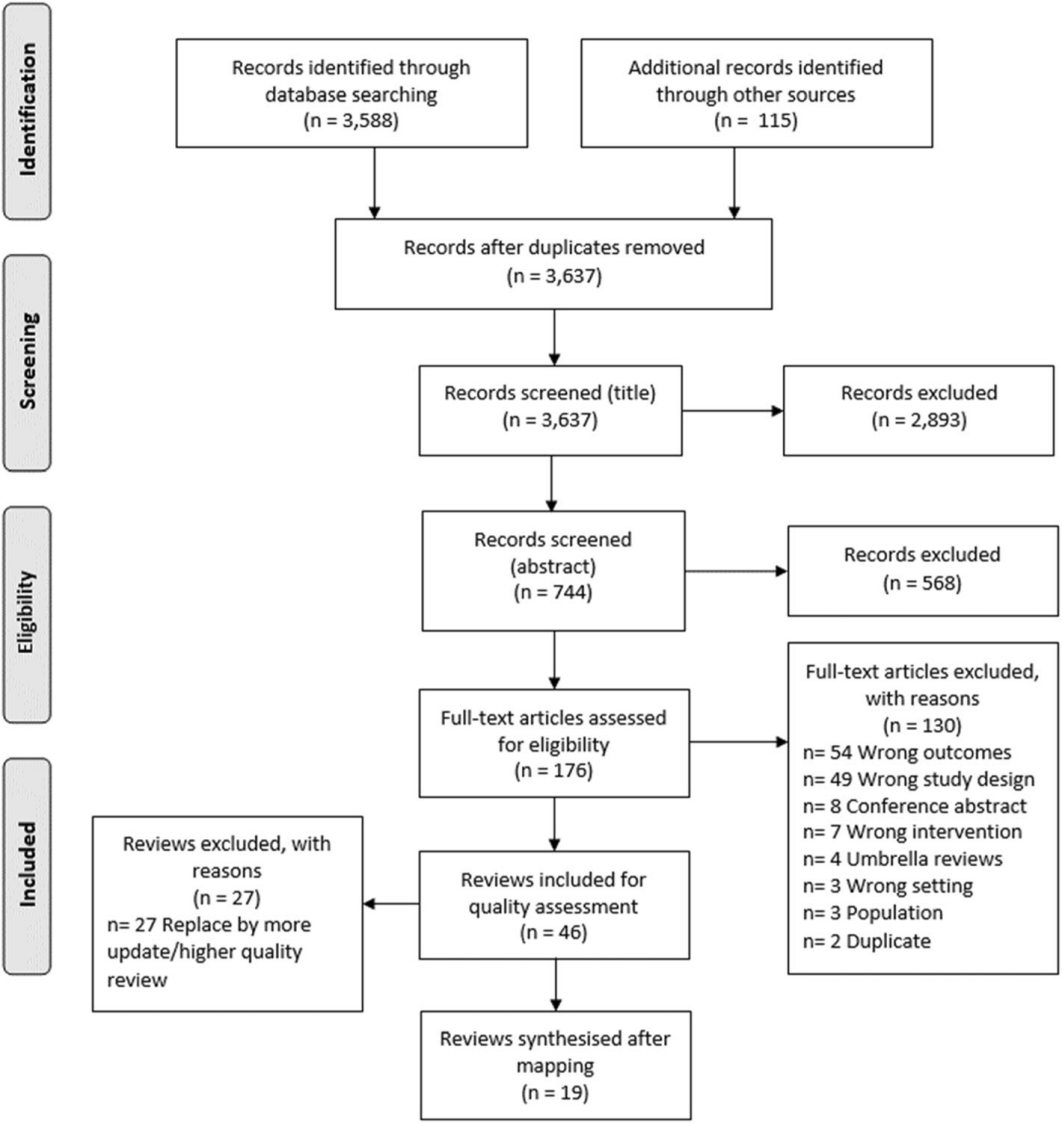
PRISMA diagram of the flow of included studies after quality assessment and mapping

A total of 3588 records were identified through database searching, with an additional 115 identified through targeted Google Scholar searches. After deduplication, and full text assessment, 46 reviews reporting the effects of 32 intervention strategies were identified. Following an assessment of review search date and quality (selecting the higher quality review where more than one review reported the effects of the same intervention strategy), 19 systematic reviews reporting the effects of 32 intervention strategies were ultimately included (Fig. 1).

### Characteristics of included reviews

Characteristics of included reviews are described in Table 1. The reviews were published between 2011 and 2020. The number of primary studies within the included reviews ranged from 13 to 120. Two reviews included only primary studies conducted in the United States (US) [52, 53] and one review was restricted to LMIC [49]. Of the 19 reviews, four included only randomised controlled trials (RCTs) or cluster RCTs, [23, 46, 54, 58] and seven undertook pooled quantitative analyses [23, 26, 44, 46, 47, 54, 55]. Eight reviews included primary studies undertaken in children and adolescents only (between 1 and 18 years), [22, 23, 46, 51, 54, 55, 57, 58] two were in adults (> 18 years) only, [50, 56] nine included all ages, [26, 43–45, 47, 49, 52, 53] and one review did not specify ages included but gave the population as workers [48].

**Table 1:** Characteristics of reviews included in the umbrella review

Author, year	Review eligibility criteria			Search strategy				No. of included primary studies
	Research design	Population	Intervention strategy	No. of data-bases	Synthesis method	Grey lit search	Search period	
Afshin et al., 2015 [43]	RCT and non-RCT; prospective cohort	Age: all ages Setting: all settings	Primary studies of policy strategies with prior evidence of effectiveness to improve diet (policies including mass media campaigns, food and menu labelling, taxation and subsidies, local built environment, school procurement policies, worksite wellness programs, and marketing standards).	1	Narrative synthesis	No	NR	14
Afshin et al., 2017 [44]	RCT and non-RCT; prospective observational	Age: all ages Setting: all settings	Primary studies of multicomponent interventions that reported the effect of the price change separately or if the price change was a major component of the intervention. The primary outcome was the change in consumption of foods and beverages; data on sales/purchase were considered a proxy for consumption.	7	Pooled quantitative synthesis	No	June 2014	26
Carter et al., 2018 [45]	RCT and non-RCT	Age: all ages Setting: any out-of-home environment (e.g. grocery stores, supermarkets, restaurants, bars, school canteens, and workplace cafeterias)	Primary studies of intervention strategies which involved the comparison of the effect of an information-based cue at point of choice on food, alcohol or tobacco selection or consumption to that of a non-information-based cue condition.	5	Narrative synthesis	No	26th Nov 2016	13
Champion et al., 2019 [46]	RCT	Age: students 11–18 years Setting: schools	Primary studies were eligible if they assessed a school-based prevention programme that was universal (i.e. delivered to all students regardless of their level of risk) and that targeted two or more of the following behaviours: alcohol use, smoking, diet, physical activity, sedentary behaviour (screen time and sitting), or sleep and were primarily delivered via eHealth methods (e.g. the internet, computers, tablets, mobile technology, or tele-health).	4	Pooled quantitative synthesis	No	1 Jan 2000–14 March 2019	16
Cornelson et al., 2015 [47]	RCT and non-RCT; cross-sectional; cohort	Age: all ages Setting: all settings	Full-text primary studies employing nationally representative data that have estimated food cross-price elasticities.	5	Pooled quantitative synthesis	Yes	28 Nov 2012	78
DeCosta et al. 2017 [22]	RCT and non-RCT	Age: 1–12 years Setting: all settings	Intervention/experimental studies measuring one or more of the following outcomes: food choice, preference, liking, intake, willingness to taste, and neophobia. Studies testing multifaceted approaches were generally not included in the review.	3	Narrative synthesis	No	Jan 2017	120
Feltner et al., 2016 [48]	RCT, non-RCT and prospective cohort studies	Age: workers, age not specified Setting: workplaces in developed countries	Primary studies of any “integrated intervention” that meets the definition of a Total Worker Health strategy (“*a strategic and operational coordination of policies, programs, and practices designed to simultaneously prevent work-related injuries and illnesses, and enhance overall workforce health and well-being.”*). Interventions may include a range of components that focus on changes in policy; organisational structure; work organisation; environmental factors; or individual worker education, counselling, training, or social support.	4	Narrative synthesis	Yes	21 Sept 2015	15
Girard et al., 2012 [49]	RCT and non-RCT	Age: children 0–59 months and/or women of reproductive age, regardless of pregnancy status Setting: households in LMIC	Agricultural intervention strategies in LMIC aimed at increasing the quantity and/or quality of household food production (i.e. gardening strategies)	5	Narrative synthesis	No	1990 – NR	36
Hendren et al., 2017 [50]	RCT and non-RCT; prospective; observational; cross-sectional	Age: employees > 18 years Setting: worksite food services	Primary studies were included if the intervention was located at workplace dining locations (e.g. cafeteria, hospital cafeteria for employees, university employee dining, and Army canteens). Employees must be adults working in public, private, government, or voluntary organisations.	4	Narrative synthesis	No	9 Aug 2016	18
Hendrie et al., 2017 [51]	RCT and non-RCT	Age: 2–12 years Setting: home and community	Primary studies were eligible for inclusion if they evaluated the effectiveness of a community-based (excluding. school) intervention strategy aimed at improving vegetable consumption, either alone or in combination with other healthy eating and lifestyle messages; and had a quantitative measure of ‘usual’ vegetable consumption separate to other food groups (such as fruit).	3	Narrative synthesis	No	2004 - June 2014	22
Hodder et al., 2020 [23]	RCT	Age: ≤ 5 years Setting: all settings	Trials of intervention strategies primarily targeting consumption of fruit, vegetables or both among children, and incorporating a dietary or biochemical assessment of fruit or vegetable consumption.	3	Pooled quantitative synthesis	No	Jan 2020	80
Hollis-Hansen et al., 2019 [52]	RCT, non-RCT and observational	Age: all ages Setting: Markets in lower-income communities	Primary studies that examined the introduction of new food retail into a lower-income community in the United States (including any Community Supported Agriculture, farmers’ market, farm stand, mobile produce market (MPM), healthy corner store, or grocery store); included F&V intake as an outcome; and determined “diet quality” as consumption of F&Vs.	3	Narrative synthesis	No	24 Aug 2018	15
Hsiao et al., 2018 [53]	RCT, non-RCT and observational	Age: all ages Setting: All settings within the United States	Primary studies based on empirical research of MPMs located in the United States; had results; analysed MPM separately from other market venues; and analysed MPMs that predominantly sold fruits and/or vegetables.	4	Narrative synthesis	No	Dec 2017	24
Langford et al., 2014 [54]	Cluster-RCT	Age: 4–18 years Setting: schools	Primary studies where randomisation took place at the level of school, district or other geographical area. HPS interventions defined as comprising the following three elements: input to the curriculum; changes to the school’s ethos or environment or both; and engagement with families or communities, or both.	20	Pooled quantitative synthesis	Yes	22 Apr 2013	67
Micha et al., 2018 [55]	RCT and non-RCT	Age: 2–18 years Setting: schools	Intervention trials that assessed the impact of school food environment policies in preschool, primary, or secondary schools on the outcomes of interest among generally healthy children.	11	Pooled quantitative synthesis	Yes	Dec 2017	91
Patnode et al., 2017 [56]	RCT and non- RCT	Age: adults, 18 years or order Setting: primary care	Primary studies that evaluated the effectiveness of behavioural intervention strategies targeting improved diet, increased physical activity, decreased sedentary time, or a combination of these targets among adults without known hypertension, dyslipidaemia, diabetes, impaired fasting glucose or glucose tolerance, or a combination of these factors.	4	Narrative synthesis	No	25 May 2016	88
Rochira et al., 2020 [57]	RCT, non-RCT and observational studies	Age: 6–13 years Setting: Schools	Primary studies with the primary intervention strategy comprised of in-school gardening projects for primary school students (age range between 6 and 13 years). We excluded studies about preschool-aged children and adolescents. We included any outcome concerning anthropometric measurements, F&V consumption or knowledge and other outcomes such as blood pressure, science achievement, physical activity, and blood samples about metabolic syndrome markers.	3	Narrative synthesis	Yes	Feb 2019	33
Rodriguez et al., 2019 [26]	RCT, cluster RCT	Age: all ages Setting: all settings	Primary studies using eHealth intervention strategies aiming to improve fruit and vegetable intake in at least one arm of the study. Studies assessing F&V intake and reporting results quantitatively; providing pre- and post-intervention means with standard deviations, or data to compute them, and sample size per each group; using true or quasi-experimental design.	6	Pooled quantitative synthesis	No	1999 to Jul 2018	19
Silveria et al., 2011 [58]	RCT	Age: 5–18 years Setting: Schools	Primary studies of nutritional education intervention strategies carried out in the school environment to reduce or prevent overweight in children and adolescents.	14	Narrative synthesis	No	5 May 2010	24

*NR* not reported

### Quality assessment of included reviews

Additional file 2 describes the outcome of the quality assessment of included reviews. Most of the reviews were of a high quality (*n* = 12), [23, 26, 44–46, 48, 50, 53–57]. Seven reviews met 34–66% of the critical appraisal criteria (CA) (i.e. scored ‘yes’) and received an overall medium quality assessment [22, 43, 47, 49, 51, 52, 58]. The primary criteria for which included reviews were downgraded were inadequate resources to search for potentially eligible primary studies (i.e. the reviews used inappropriate databases, searched two or fewer databases, or did not search the grey literature search) (CA4) (9 reviews).

### Effectiveness of fruit and vegetable intervention strategies

The effects of 32 intervention strategies were synthesised from the 19 included reviews. There was evidence supporting the effectiveness of 19 of the 32 intervention strategies (Table 2). Among reviews reporting meta-analyses of the effects on combined fruit and vegetable intake, effect sizes were largest for school-based strategies that provided free (or reduced price) fruits and vegetables, or increased their in-school availability (+ 0.28 serves); strategies that targeted child feeding practices in childcare services, and the home and family environment; and computer-based and SMS delivered intervention strategies. All of these intervention strategies improved intake by between 0.41 and 0.63 of a standard deviation (Additional file 3). Across reviews, intervention strategies that sought to increase the physical availability of fruits and vegetables in settings or communities appeared to be broadly effective, including those in school and workplace cafeterias, school gardens and domestic home-based agricultural settings.

**Table 2:** Effects of interventions strategies synthesised by included systematic reviews on measures of fruit and vegetable intake or purchase

Author, year of publication	Intervention strategy	NOURISHING policy domain and policy area	Primary study quality assessment	Findings	Effect Summary
**School based intervention strategies**					
Micha et al., 2018 [55]	Providing (for free or at reduced cost) or increasing the availability (e.g. in cafeterias) of F&V	Domain: Food environment (FE) Policy area: Economic tools to address affordability and purchase incentives	Five studies had an overall quality score of five; five studies had a score of four; five studies had a score of three; and four studies had a score of two. ^a^	Fruit: Increased intake by 0.27 servings/day (95%CI 0.17, 0.36; *p* = 0.000; I^2^ 78.3%; *n* = 15 primary studies) Vegetables: Increased intake (marginally) by 0.04 servings/day (95%CI 0.01, 0.08; *p* = 0.221; I^2^ 23.4%; *n* = 11 primary studies) Combined: Increased F&V intake by 0.28 servings/day (95%CI: 0.17, 0.4; p = <.001; I^2^ 90.2%; *n* = 16 primary studies)	Y
Micha et al., 2018 [55]	Food standard policies	Domain: FE Policy area: Offer healthy food and set standards in public institutions and other specific settings	Three studies had an overall quality score of two; one study had a score of three; and one study had a score of four.^a^	Fruit: increased by 0.76 servings/day (95%CI: 0.37, 1.16; p = NR; I^2^ NR; n = 2 primary studies) Vegetable: Non-significant trend toward increased intake of 0.30 servings/day (95%CI: −0.001, 0.59; p = NR; I^2^ NR; *n* = 2 primary studies) Combined: nonsignificant trends toward increased intake of F&V intake of 0.12 servings/day (95%CI: − 0.08, 0.31; p = NR; I^2^ NR; n = 5 primary studies)	Y
Rochira et al., 2020 [57]	School Gardens	Domain: Behaviour Change Communication (BCC) Policy area: Give nutrition education and skills	Of the 15 studies assessed using the Cochrane Tool for Quality Assessment 3 were assessed as good quality while 12 scored fair quality. ^b,o^ Of the 17 observational studies rated using the STROBE tool, 4 scored intermediate quality and 13 scored good quality. ^b, o^	“The selected articles demonstrate that children increased F&V daily/weekly intake” (*n* = 20 primary studies)	Y
Silveria et al., 2011 [58]	Circular/ nutrition education	Domain: BCC Policy area: Give nutrition education and skills	Of the 16 primary studies that measured fruit and/or vegetable consumption, four were rated as “quality level A”, nine were “quality level B”, and three were “quality level C”. ^c^	“Of the 12 studies that adopted at least two of the three most common components (activities in the classroom, parental development and school feeding policy), 10 presented results that confirm the effectiveness of nutritional education intervention strategies in schools for increased consumption of F&V among children and adolescents.”	Y
Champion et al., 2019 [46]	School eHealth	Domain: BCC Policy area: Give nutrition education and skills	The quality of evidence for screen time and fruit and vegetable outcomes immediately after the intervention was deemed to be moderate. ^c^	eHealth school-based multiple health behaviour change intervention strategies led to a small but significant increase in fruit and vegetable intake immediately after the intervention (Standardised mean difference (SMD) = 0.11; 95%CI: 0.03, 0.19; *p* = 0.007; I^2^ 42% n = 7 primary studies). Effects on F&V intake were not sustained at follow-up (SMD 0.07; 95%CI: − 0.01, 0.15; *p* = 0.07; I^2^ 52% n = 6 primary studies)	?
Langford et al., 2014 [54]	Health Promoting schools	Domain: FE, BCC Policy area: FE: Offer healthy food and set standards in public institutions and other specific settings BCC: Give nutrition education and skills	GRADE assessment for 23 nutrition trials was ‘Low’. ^d, o^	Nutrition only intervention strategies were effective on average at increasing reported F&V intake among students (SMD 0.15; 95%CI: 0.02, 0.29; p = NR; I^2^ = 83%; n = 9 primary studies).	Y
DeCosta et al., 2017 [22]	Cooking lessons/classes (i.e. not solely school-based)	Domain: BCC Policy area: Give nutrition education and skills	No formal quality assessment.	“Evidence suggests that cooking classes may positively change intake and preference for vegetables and that the effect might be mediated by tasting new F&V. However, based on the little evidence currently available, no conclusions regarding best practise can be made. Additionally, long-term effects have not been investigated.” (n = 6 primary studies)	?
**Childcare based intervention strategies**					
Hodder et al., 2020 [23]	Child feeding intervention strategies delivered by childcare providers	Domain: BCC Policy area: Give nutrition education and skills	GRADE for child feeding intervention strategies was ‘Low’.^d^, ^o^	Child feeding intervention strategies for childcare providers improved fruit and/or vegetable intake of children: SMD 0.63 (95%CI 0.23, 1.03; p = 0.002; I^2^ 81%; n = 8 primary studies)	Y
Hodder et al., 2020 [23]	Parent nutrition education intervention strategies delivered in childcare	Domain: BCC Policy area: Give nutrition education and skills	GRADE for parent nutrition education intervention strategies was ‘Very Low’.^d, o^	Parent nutrition education did not significantly improve child fruit and/or vegetable intake: SMD 0.43 (95%CI −0.27, 1.13; *p* = 0.23; I^2^ 84%; n = 2 primary studies)	?
Hodder et al., 2020 [23]	Multicomponent interventions	Policy domain: BCC Policy area: Give nutrition education and skills	GRADE for multi-component interventions was ‘Moderate’.^d, o^	Multi-component interventions delivered in childcare did not significantly improve child fruit and/or vegetable intake: SMD 0.21 (95%CI − 0.07, 0.49; *p* = 0.15; I^2^ 78%; n = 5 primary studies)	?
**Parent and family home-based intervention strategies**					
Hodder et al., 2020 [23]	Nutrition education	Domain: BCC Policy area: Give nutrition education and skills	GRADE for parent nutrition education interventions was ‘Very Low’.^d, o^	Parent nutrition education interventions delivered at home did not improve child F&V intake: SMD = 0.07 (95%CI −0.14, 0.27; *p* = 0.52; I^2^ 68%; n = 5 primary studies)	?
Hodder et al., 2020 [23]	Child feeding intervention strategies	Domain: BCC Policy area: Give nutrition education and skills	GRADE for child feeding intervention strategies was ‘Low’.^d, o^	Child feeding intervention strategies targeting parents significantly improved child F&V intake: SMD = 0.46 (95%CI: 0.13, 0.79; p = 0.007; I^2^ 68%; n = 4 primary studies)	Y
**Workplace-based intervention strategies**					
Hendren et al., 2017 [50]	Cafeteria intervention strategies	Domain: FE, BCC Policy area: FE: Standards in other specific locations; Nutrition label standards and regulations on the use of claims and implied claims on food; Use economic tools to address food affordability and purchase incentives BCC: Inform people about food and nutrition through public awareness	Of the 14 primary studies reporting significant results, five were rated as high-quality, six as moderate-quality, and three as low-quality.^k^	“There appears to be a moderately strong association toward a positive impact of cafeteria intervention strategies to increase fruit and/or vegetable consumption. Of the 18 studies in the review, 13 reported a statistically significant increase, one reported a significant decrease, three reported mixed results, and one did not assess a change in consumption.” Six studies assessed long-term follow-up and reported mixed results	Y
Feltner et al., 2016 [48]	Total Worker Health (TWH) program (“target e protection from work-related safety and health hazards with prevention efforts)	Domain: BCC, FE Policy area: BCC: Give nutrition education and skills FE: Offer healthy food and set standards in public institutions and other specific settings	All three RCTs that measured changes in fruit and vegetable intake, were rated as have a low strength of evidence.^n^	“Three RCTs (all from the same research team) … measured changes in fruit and vegetable intake among US manufacturing or construction workers who were randomly assigned to a multicomponent integrated intervention or no intervention. Evidence from these three RCTs supported the effectiveness of TWH intervention strategies compared with no intervention for improving fruit and vegetable consumption over 26 to 104 weeks.”	Y
**Primary care intervention strategies**					
Patnode et al., 2017 [56]	Counselling - individual or group; via in-person, telephone, web-based, text message, and/or print mailing	Domain: BCC Policy area: Nutrition advice and counselling in healthcare settings	Of the 26 primary studies reporting fruit and vegetable intake, three were rated as ‘good’ quality and the remaining 23 were rated as ‘fair’ quality.^e^	“… Between-group differences in the mean change of fruit and vegetable intake ranged from −0.2 servings/day (favouring the control group) to 2.2 servings/day (favouring the intervention group) at 6 months to 1 year of follow up. All six trials that focused dietary messages exclusively on increased fruit and vegetable intake found statistically significantly greater benefit among intervention versus control participants” (*n* = 26 primary studies)	Y
**Community-based individual and group based programs**					
Hendrie et al., 2017 [51]	After school community-based nutrition education and skill based programs for children and/or families	Domain: BCC Policy area: Give nutrition education and skills	Three studies were rated as weak quality and one was rated as moderate quality.^i, o^	Of the four primary studies assessing children’s vegetable intake, two studies assessed longer-term effectiveness (6 months) on children’s vegetable intake. One of two trials of after school programs were effective in the longer-term (6 months)	?
**eHealth intervention strategies**					
Rodriguez et al., 2019 [26]	Any eHealth intervention (i.e. includes the use technology to deliver and enhance health services and information)	Domain: BCC Policy area: Give nutrition education and skills	Most primary studies were assessed as being fair quality (n = 12), five as good quality, and two as poor quality.^f^	Overall e-Health intervention strategies improved fruit and/or vegetable intake: SMD/Hedge’s *g =* 0.26 (SE 0.05; 95%CI 0.17, 0.35; *p* < 0.001; I^2^ NR; *n* = 19 primary studies)	Y
Rodriguez et al., 2019 [26]	Computer-based intervention strategies	Domain: BCC Policy area: Give nutrition education and skills	Most primary studies were assessed as being fair quality (n = 12), five as good quality, and two as poor quality.^f^	Computer-based intervention strategies improved fruit and/or vegetable intake: SMD = 0. 44 (SE 0.08; 95%CI NR; *p* < 0.001; I^2^ NR; n = 3 primary studies)	Y
Rodriguez et al., 2019 [26]	SMS intervention strategies	Domain: BCC Policy area: Give nutrition education and skills	Most primary studies were assessed as being fair quality (*n* = 12), five as good quality, and two as poor quality.^f^	SMS intervention strategies improved fruit and/or vegetable intake: SMD/Hedge’s *g =* 0.41 (SE 0.10; 95%CI 0.21, 0.61; *p* < 0.01; I^2^ NR; *n* = 3 primary studies)	Y
Rodriguez et al., 2019 [26]	Internet-based intervention strategies	Domain: BCC Policy area: Give nutrition education and skills	Most primary studies were assessed as being fair quality (n = 12), five as good quality, and two as poor quality.^f^	Internet-based intervention strategies improved fruit and/or vegetable intake: SMD/Hedge’s g 0.19 (SE 0.05; 95%CI 0.09, 0.29; *p* < .001; I^2^ 42; n = 9 primary studies)	Y
Rodriguez et al., 2019 [26]	CD-ROM, intervention strategies	Domain: BCC Policy area: Give nutrition education and skills	Most primary studies were assessed as being fair quality (n = 12), five as good quality, and two as poor quality.^f^	CD-ROM intervention strategies did not significantly improve F&V intake: SMD/Hedge’s g 0.09 (SE 0.10; 95%CI NR; *p* > .05; I^2^ NR; n = 2 primary studies)	?
Rodriguez et al., 2019 [26]	Mobile apps, intervention strategies	Domain: BCC Policy area: Give nutrition education and skills	Most primary studies were assessed as being fair quality (n = 12), five as good quality, and two as poor quality.^f^	Mobile-based (app) intervention strategies did not significantly improve F&V intake: SMD/Hedge’s g 0.13 (SE 0.15; 95%CI NR; p > .05; I^2^ NR; n = 1 primary study)	?
Rodriguez et al., 2019 [26]	Video game intervention strategies	Domain: BCC Policy area: Give nutrition education and skills	Most primary studies were assessed as being fair quality (n = 12), five as good quality, and two as poor quality.^f^	Video game intervention strategies did not significantly improve F&V intake: SMD/Hedge’s g 0.08 (SE 0.23; 95%CI NR; p > .05; I^2^ NR; n = 1 primary studies)	?
**Mass media campaigns**					
Afshin et al., 2015 [43]	Media	Domain: BCC Policy area: Inform people about food and nutrition through public awareness	No formal quality assessment.	“Several studies suggest potential effectiveness of mass media campaigns as a stand-alone intervention. These have shown temporal improvements in consumption of specific dietary factors, especially increased F&V …” (n = 5 primary studies)	Y
**Choice architecture**					
DeCosta et al., 2017 [22]	Choice Architecture strategies in school food service, tuck shops and vending machines	Domain: BCC Policy area: Public awareness, mass media and informational campaigns and social marketing on healthy eating	No formal quality assessment.	“In school settings, choice architecture and nudging have been shown to positively increase selection and overall consumption of fruits and vegetables in the short term. However, evidence for long-term benefits is sparse.” (n = 7 primary studies)	?
Carter et al., 2018 [45]	Information based cues	Domain: BCC Policy area: Inform people about food and nutrition through public awareness	Of the three primary studies undertaken in supermarkets, two were assessed as high risk of bias and the remaining study at unclear risk.^c, o^	“In relation to supermarkets, two of the three included studies observed significant increase in sales of fruit and vegetables.” (n = 3 primary studies)	?
**New food retail opportunities**					
Hsiao et al., 2019 [53]	Mobile Produce markets (MPM)	Domain: Food system Policy area: Harness supply chain and actions across sectors to ensure coherence with health	All four primary studies assessing fruit and/or vegetable intake received a quality score of two. ^j^	Positive associations were observed for all five studies that assessed the relationship between Mobile Produce Markets (MPM) use and fruit and/or vegetable intake. “Overall, intervention primary studies (n = 4) found consistent increases in reported vegetable intake and in combined reported fruit and vegetable intakes during the study periods among MPM users, with greater changes observed in vegetable intake.”	Y
Hollis-Hansen et al., 2019 [52]	Farmers markets	Domain: Food System (FS) Policy area: Harness supply chain and actions across sectors to ensure coherence with health	No formal quality assessment.	“Two of five primary studies of new farmers markets reported improvements in F&V intake ranging from −0.70 to + 0.70 cups/day and Cohen’s *d* effect sizes ranging from 0.15 to 0.38. “ “The remaining three primary studies report findings graphically or with frequencies, and these suggest improvements in F&V consumption although insufficient data are available to draw a conclusion.”	?
Hollis-Hansen et al., 2019 [52]	Retail supermarkets	Domain: FS Policy area: Harness supply chain and actions across sectors to ensure coherence with health	No formal quality assessment.	“None of the four retail supermarket primary studies reported a positive impact on F&V consumption. Two of the primary studies reported statistically significant inverse findings that suggest the introduction of a new retail supermarket may have decreased F&V consumption, with a third study also reporting an inverse finding that was not statistically significant.”	X
**Agricultural intervention strategies**					
Girard et al., 2012 [49]	Household food production	Policy domain: Food system Policy area: Community food production (directly mapped to home-based gardening strategies)	GRADE for the five primary studies assessing fruit and vegetable intake was low to very low.^d^	“In general home gardening strategies both with and without animal production improved … the consumption of vitamin A (VA)-rich fruits and vegetables. Findings for VA-rich foods were consistent regardless of whether the home gardening strategy included an animal production component.” (n = 5 primary studies)	Y
**Food pricing intervention strategies**					
Afshin et al., 2017 [44]	Subsidy	Domain: FE Policy area: Use economic tools to address food affordability and purchase incentives	Used CDC Community Guide grading system: ‘Strong Evidence, Strongly Recommended grade’.^g^, ^P^	A 10% price decrease increased consumption of fruits and vegetables by 14% (95%CI: 11 to 17%). (n = 9 primary studies)	Y
Cornelson et al., 2015 [47]	Tax/price increase	Domain: FE Policy area: Use economic tools to address food affordability and purchase incentives	Of the 24 primary studies with fruit and vegetable outcomes, 41.2% were classified as ‘replicable’.^h^	Low income countries −10% increase in the price of F&V = decrease in F&V 7.2%; *p* < .001 − 10% increase in the price of meat = increase in F&V 0.05%; p = not significant (NS) − 10% increase in the price of Fish = decrease in F&V 0.14%; p = NS − 10% increase in the price of Dairy = decrease in F&V 0.01%; p = NS − 10% increase in the price of Cereals = increase in F&V.65%; *p* < .10 − 10% increase in the price of Fats & oils = decrease in FV 0.14%; p = NS − 10% increase in the price of sweets = increase in F&V 1.12%; *p* < .001 Middle income countries − 10% increase in the price of F&V = decrease in F&V 6.5%; *p* < .001 − 10% increase in the price of meat = decrease in F&V 0.26%; p = NS − 10% increase in the price of Fish = decrease in F&V 0.79%; *p* < 0.05 − 10% increase in the price of dairy = decrease in F&V 0.58%; *p* < 0.05 − 10% increase in the price of cereals = increase in F&V 0.07%; p = NS − 10% increase in the price of fats & oils = decrease in F&V 0.39%; p = NS − 10% increase in the price of sweets = increase in F&V 0.34%; p = NS High income countries − 10% increase in the price of F&V = decrease in F&V 5.3%; *p* < .001 − 10% increase in the price of meat = increase in F&V 0.02%; p = NS − 10% increase in the price of Fish = increase in F&V 0.10%; p = NS − 10% increase in the price of dairy = decrease in F&V 0.30%; *p* < .001 − 10% increase in the price of cereals = increase in F&V 0.48%; p = NS − 10% increase in the price of fats & oils = decrease in F&V 0.33%; p = NS − 10% increase in the price of sweets = increase in F&V 0.60; *p* < .001	Y/X

^a^Tool not reported. Scores from 0 to 3 were considered lower quality and 4 to 5 higher quality

^b^Cochrane Tool for Quality Assessment and the Strengthening the Reporting of Observational Studies in Epidemiology (STROBE)

^c^A modified version of the Quality Assessment Tool for Quantitative Studies, developed by the Effective Public Health Practice Project (EPHPP) and GRADE. Primary studies were categorized as: A, high quality (EPHPP ≥4 and GRADE ≥ 3); B, regular quality (EPHPP ≥4 and GRADE = 2); or C, low quality (EPHPP ≤3 or GRADE ≤ 1)

^d^Cochrane tool for assessing risk of bias and GRADE

^e^Cochrane tool for assessing risk of bias

^f^USPSTF design-specific criteria. Each primary study was assigned a rating of “good” “fair” or “poor” based on the methodological quality

^g^Cochrane tool for assessing risk of bias. Agency for Healthcare Research and Quality Standards used to categorize primary study quality as good, fair, or poor

^h^Tool not reported. Quality assessment based on 5 criteria. Strength of evidence graded using Center for Disease Control and Prevention Community Guide

^i^Tool not reported. Quality assessment based on whether the description was sufficient for replication of the study

^j^Effective Public Health Practice Project (EPHPP) quality assessment tool

^k^Mixed Methods Appraisal Tool (MMAT) version 2011. An overall quality score based on number of criteria met (0–4)

^l^A quality and bias assessment tool created with scores presented by quartile (25th quartile = low quality; 25th–75th = moderate quality; 75th = high quality)

^m^Primary study quality was independently assessed by two authors according to the Jadad Score. Studies were scored according to the presence of three key methodological features of clinical trials, specifically randomisation, masking, and accountability of all participants, including withdrawals

^n^Strength of evidence rated as high, moderate, low, or insufficient according to the AHRQ Methods Guide for Effectiveness and Comparative Effectiveness Reviews

^o^The quality assessment refers to assessments undertaken on all included primary studies and may not represent the studies included in the fruit and vegetable outcome reported

^P^Graded the strength evidence also using tools from i) American Heart Association; ii) U.S. Preventive Services Task Force; iii) Centers for Disease Control and Prevention Community Guide. Reported

Y Overall judged as likely to have a positive effect on measures of fruit and/or vegetable intake based on assessment of statistical significance of point estimates in meta-analyses, or summary statements for narrative reviews

X Overall judged as likely to have a negative impact on measures of fruit and/or vegetable intake based on assessment of statistical significance of point estimates in meta-analyses, or summary statements for narrative reviews

? Overall the effects of the intervention strategies are uncertain

Strategies that focused on nutrition awareness and education and skill development reported more mixed effects. For example, review evidence suggests that school-based nutrition education and curricula, mass media campaigns and nutrition counselling in primary care were effective in improving intake. More equivocal were review findings regarding the effects of nutrition education focused strategies delivered via childcare services, targeting parents and the home environment, or undertaken as part of individual and group-based community programs. Finally, although reductions or increases in the price of fruit and vegetable products were found to enhance (for price reductions of subsidies) or reduce (for price increases or taxes) fruit and vegetable intake, the long-term effects of other strategies that could be implemented within retail environments (e.g. choice architecture strategies) were mixed. Similarly, there was some evidence to support the introduction of mobile produce markets as a strategy to improve intake of fruits and vegetables among market users, but the introduction of new supermarkets (which offer both fresh and packaged foods) may reduce intake. The rest of this section outlines the effects of intervention strategies reported in the included reviews, grouped by setting where appropriate.

#### Setting-based interventions

##### School-based intervention strategies

Six included systematic reviews reported the effects of seven school-based intervention strategies. In one of these reviews**,** which assessed the effects of cooking lessons, four of six primary studies were undertaken in the school setting (the other two occurred in the home) [22]. Five of the seven school-based intervention strategies were reported to be effective on at least one measure of fruit, vegetable, or combined fruit and vegetable intake, including providing free (or reduced price) fruits or vegetables, or increasing their within-school availability [55]; implementing food standard policies [55]; providing school gardens [57]; providing nutrition education and curricula [58]; and applying the Health Promoting Schools framework [54]. With the exception of nutrition education, effective intervention strategies were those targeting policy or modification of the school food environment. Among the reviews reporting meta-analyses, effect sizes were particularly high for food standard policies, which increased fruit intake by 0.76 serves/day (95% confidence interval [CI]: 0.37, 1.16; p = not reported [NR]; *n* = 2 primary studies) [55]. Reviews reporting on strategies focusing on communication and behaviour change via knowledge or skill acquisition – for example, cooking lessons [22] and school-based eHealth [46] – suggest that such strategies are effective immediately following the intervention, but in the long-term the effects are equivocal (*n* = 6 primary studies).

##### Childcare-based intervention strategies

One review, reporting the effects of three childcare-based intervention strategies, was included. The review of randomised trials reported a significant pooled effect on child fruit and vegetable intake for child-feeding interventions delivered by childcare staff (standardized mean difference [SMD] = 0.63; 95%CI: 0.23, 1.03; *p* = 0.002; *n* = 8 primary studies), but not for parent nutrition education intervention strategies delivered through childcare, or for multi-component childcare intervention strategies.

##### Parent and family home-based intervention strategies

One review, reporting the effects of two parent or family home-based intervention strategies, was included. A review of randomised trials found intervention strategies targeting child-feeding practices of parents (i.e. repeated food exposure) were effective in the short term (< 12 months) on fruit and vegetable intake (SMD = 0.46; 95%CI: 0.13, 0.79; *p* = 0.007; *n* = 4 primary studies); however, parent nutrition education strategies were not effective [23].

##### Workplace-based intervention strategies

Two reviews reported the effects of two workplace-based intervention strategies, the effects of which were mixed. In one review, strategies targeting workplace cafeterias (with or without nutrition education) [50] reported consistent improvements (from 13 of 18 primary studies) in fruit and vegetable intake post-intervention; however, evidence of effect at longer-term follow-ups (> 12 months) was equivocal. The other review examined intervention strategies to improve workplace health and safety integrated with health promotion interventions to advance worker well-being [48]. It cited evidence from three RCTs that demonstrated the effectiveness of such intervention strategies in improving fruit and vegetable intake over 26–104 weeks.

##### Primary-care intervention strategies

One included review [56] examined the effects of primary care behavioural counselling interventions, either alone or as part of a larger multicomponent intervention on a range of behavioural risk factors for coronary heart diseases. The intervention strategies were delivered via a range of formats (e.g. face to face, telephone or web) and reported effect sizes ranging from − 0.2 to 2.2 servings per day for fruits and vegetables (*n* = 26 primary studies). Heterogeneity of the primary studies precluded meta-analysis, but the review concluded that nutrition intervention strategies based in primary care generally resulted in small increases in fruit and vegetable intake.

##### Community-based individual and group-based programs

One review examined community-based after-school programs [51]. It found that behavioural skills training and education with children and/or parents were effective in the short term (for vegetable intake only); however, the longer term effects (> 6 months) on measures of fruit or vegetable intake were mixed. The effects sizes on these measures ranged from an SMD of 0.18 to 0.25 (*n* = 4 primary studies).

#### Non-setting-based interventions

##### eHealth intervention strategies

One review reported the effects of various eHealth intervention strategies [26]. An overall pooled analysis of all such strategies found they were effective in improving fruit and vegetable intake in adults and children (SMD = 0.26; 95%CI: 0.17, 0.35; *p* < 0.001; I^2^ not reported; *n* = 19 primary studies). Subgroup analyses reported significant effects for computer-based, SMS and Internet-based intervention strategies, but not for those delivered via CD-ROM, mobile apps or video games.

##### Mass media intervention strategies

A narrative synthesis of five primary studies of mass media intervention strategies targeting nutrition behaviours suggested the potential effectiveness of these as stand-alone approaches in improving the consumption of fruits and vegetables in adults and young people [43].

##### Choice architecture

Two narrative reviews that examined the effects of choice architecture strategies reported mixed findings. One review did not disaggregate the effects of specific strategies, but found that evidence of the long-term effects of choice architecture intervention strategies (e.g. food signage, changes to food description, presentation and verbal prompts in cafeterias) was unclear overall (*n* = 7 primary studies) [22]. The other review found limited but supportive evidence from two of three primary studies that fruit and vegetable sales were improved by information-based cues in supermarket settings (excluding labels of nutritional content or nutritional values, and those delivered on TV or internet, or that were interactive) (*n* = 3 primary studies) [45].

##### New food retail opportunities

Two reviews reported the effects of the introduction of three types of new food retail outlets. These reviews, which were restricted to primary studies undertaken in the US, [52, 53] suggested that intake of fruits and vegetables may be increased by the introduction of new retail opportunities that predominately provide access to fresh produce (e.g. fruits and vegetables), but not by opportunities that increase access to a broader range of fresh and packaged foods. Specifically, it was suggested that mobile produce markets increase fruit and vegetable intake among market users (*n* = 4 primary studies), [53] farmers markets introduced in lower income communities have mixed effects (− 0.70–0.70 cups per day), [52] and new food retail markets (supermarkets) may have a detrimental impact on fruit and vegetable intake (4 primary studies) [52].

##### Agricultural intervention strategies

One review [49] of randomised and non-randomised trials in LMIC examined the effects of household food production intervention strategies on the nutrition and health outcomes of women and children (*n* = 5 primary studies). It concluded that household gardens, with or without an animal production component, improved intake of fruits and vegetables rich in vitamin A rich.

##### Food pricing intervention strategies

Two reviews reported the effects of two fiscal intervention strategies: price reductions and price increases. One review [44] pooled data from nine interventional and prospective cohort studies, and found that a 10% decrease (or subsidy) in the price of fruits and vegetables increased their consumption by 14% (95%CI: 11 to 17%) (*n* = 9 primary studies). The other review [47] found that a 10% increase (e.g. tax) was associated with reductions in consumption of fruits and vegetables of 7.2, 6.5 and 5.3% in low-, medium- and high-income countries, respectively (*n* = 24 primary studies). Price increases on other food types, such as sweets, were found to have a marginal positive impact on consumption of fruits and vegetables [47].

### Review evidence gaps

Table 3 illustrates how the intervention strategies synthesised in the included systematic reviews align with policy action areas suggested in the WCRF NOURISHING framework (represented by the shaded cells). The intervention strategies could be mapped across all three broad framework domains (food environment, food system and behaviour change communication) and across seven of the 10 broad policy areas. However, they covered just 14 of the 65 specific sub-policy areas in the framework (see Table 3); thus, systematic review evidence is not available for most of the recommended sub-policy areas. Intervention strategies were mapped most frequently to sub-policy areas of the behaviour change and communication domain of the framework. Very little review evidence described the effects of intervention strategies in the food system domain. Nonetheless, the included reviews provided evidence supporting the effectiveness of intervention strategies in most of the sub-policy areas for which reviews could be mapped.

**Table 3:**
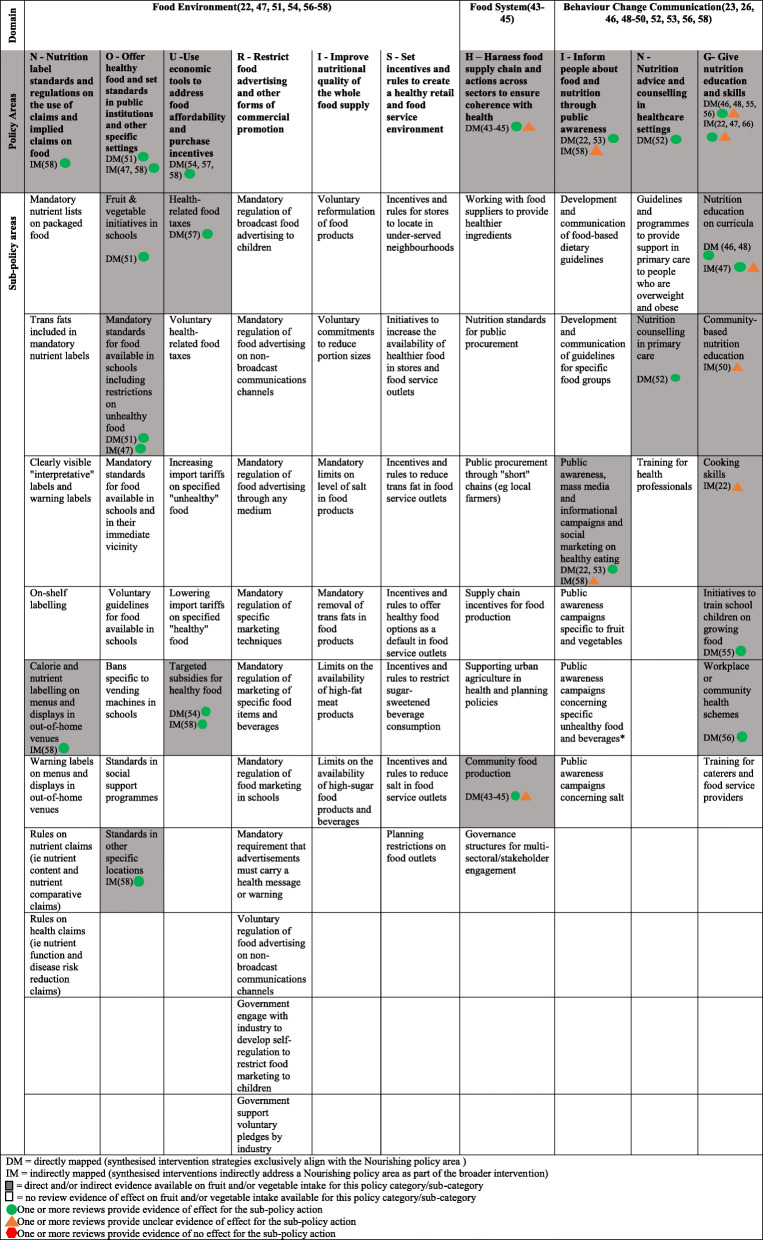
Evidence from systematic reviews synthesised in the umbrella review mapped to the NOURISHING Framework

All the intervention strategies synthesised by the included reviews that mapped to the ‘food environment’ domain were reportedly effective in improving fruit and vegetable intake, including those within the policy areas of ‘Nutrition label standards and regulations’ and ‘Offering healthy food and set standards in public institutions and other settings’, such as schemes to increase the availability of fruits and vegetables in schools. There was also direct evidence from systematic reviews regarding the impact of interventions in the policy area of ‘Use of economic tools to address food affordability and purchase incentives’. This evidence supported sub-policy areas such as the use of food taxes [47] on foods such as sweets (to reduce intake), and targeted subsidies for fruits and vegetables [43].

In contrast to intervention strategies mapped to the food environment domain, the strategies mapped to the food system and behaviour change communication domain did not always have a beneficial effect. Within the ‘food system domain’, evidence from systematic reviews could be mapped directly to just one sub-policy area – ‘Community food production’ – for which reviews found that home gardens in LMIC improved fruit and vegetable consumption, whereas evidence of the benefits of introducing fresh-produce markets was less certain [49].

Within the ‘Behaviour change communication domain’, direct evidence of a beneficial effect was identified within the policy areas of ‘Informing people about food and nutrition through public awareness’ (specifically, the use of mass media campaigns) [43] and ‘nutrition advice and counselling in healthcare settings’ (specifically, nutrition counselling in primary care) [56]. Within the policy area of ‘Give nutrition education and skills’, systematic review evidence could be directly mapped to nutrition education on curricula, [58] initiatives to train school children on growing food [57] and workplace health schemes, [48] each of which was reported as being effective in improving fruit and vegetable intake. However, within this policy area, evidence of the beneficial effects of community-based nutrition education [51] and cooking skills [22] was equivocal.

## Discussion

This umbrella review sought to consolidate the global evidence base from systematic reviews regarding the effectiveness of intervention strategies to improve fruit and vegetable intake. It identified several strategies with evidence of a beneficial impact, mapped to the WCRF NOURISHING framework, including those undertaken in community settings (e.g. schools, childcare services and workplaces), eHealth and mass media, household food production strategies, and fruit and vegetable subsidies. The findings indicate that policy-makers and practitioners have a range of effective options to improve population level intake of fruits and vegetables. Nonetheless, the evidence on the effects of intervention strategies represented only a fraction of the options suggested by comprehensive nutrition frameworks; thus, many recommended nutrition actions have not been the subject of systematic synthesis to determine their specific impact on fruit and vegetable intake.

Intervention strategies for which systematic reviews reported an effect were predominately focused on the food environment and behaviour change communication domains of the WCRF NOURISHING framework, and most of these reviews reported the effects of setting-based interventions (particularly schools). Encouragingly, school-based fruit and vegetable intervention strategies are frequently undertaken by governments, and this umbrella review provides further evidence to support investment in such initiatives. However, the included reviews did not assess the impacts of most of the framework sub-category areas, and none of them reported the effects of intervention strategies within the broad categories of ‘Restrictions on food advertising and commercial promotion’ or ‘Improving the quality of the whole food supply’. Strategies such as nutrition labelling and food reformulation can improve energy or macronutrient intake, [59, 60] and are often recommended as pillars of public health approaches to prevent chronic diseases [61]. Although these types of strategy may not directly target fruit and vegetable intake, they could conceivably increase it. The conduct of primary studies and systematic reviews to address these identified evidence gaps is warranted, to provide further evidence to justify the application of such strategies.

Pricing strategies have been suggested as a powerful determinant of dietary behaviour. Their use to improve public health nutrition has been recommended by the UN General Assembly at a High-Level Meeting on Non-Communicable Diseases [62]. Indeed, countries such as Denmark, Hungary, Mexico and the US have introduced fiscal strategies targeting food and drink items [63]. The findings of this review provide evidence to support the influence of changes in the relative price of fruits and vegetables on their intake, particularly in LMIC. Specifically, reducing the cost of fruits and vegetables by 10% (through the introduction of subsidies) can increase intake by 14%, while price increases of the same magnitude will reduce consumption by 5–7%. Taxes on other food products (e.g. sweets) were also found to increase fruit and vegetable intake, albeit marginally, suggesting that raising the price of unhealthy foods may contribute to healthier diets more broadly [47]. Introducing both subsidies and taxes may represent a particularly potent tool for increasing fruit and vegetable consumption. Modelling undertaken in Australia, for example, suggests that such an approach can yield significant health gains, and cost savings from a health system perspective [63]. Fiscal measures, however, need to be carefully designed to ensure that they do not exacerbate inequities, given their disproportionate impact on the most financially disadvantaged population groups.

Access to fruits and vegetables is a critical determinant of intake [64]. This umbrella review confirmed that school-based strategies to improve the availability to fruit and vegetable products are an effective way to increase child intake of these products. More broadly, research suggests that improving the availability of healthy foods within schools, [55] workplace food services or cafeterias, [50] or other food outlets [65] is associated with greater intake of these foods. However, improving availability may be particularly challenging in countries where the supply of fruits and vegetables is limited [13]. Within such settings, choice architecture, coupled with strategies to increase the accessibility and palatability of fruits and vegetables, represent promising avenues to further increase intake and reduce wastage, although more research is required to confirm the effects of these intervention strategies in the longer term [22].

Interestingly, the umbrella review found mixed effects reported from efforts to improve the physical availability of fruits and vegetables through new food retail outlets, such as supermarket grocery stores, mobile produce markets and farmers markets. Farmers and mobile produce markets were suggested to be beneficial in improving fruit and vegetable intake because they provide access to fresh produce, including fruits and vegetables. In contrast, the introduction of new retail supermarkets appeared to have a detrimental effect on fruit and vegetable intake. Also supermarkets may increase the availability of fruits and vegetables, they also increase access to less healthy foods and exposure to unhealthy food marketing, which may explain, in part, these apparently contradictory findings.

The findings of the review should be considered in the context of several limitations. First, as an umbrella review, the study was restricted to the analyses reported in the included reviews, and did not re-examine or analyse primary studies. Thus, the identified gaps in the evidence base for some intervention strategies may reflect the lack of systematic reviews of these strategies rather than the absence of primary studies. Second, it is likely that there is some duplication of primary studies in the syntheses of the effects of intervention strategies reported by the included reviews, particularly among reviews reporting strategies conducted in the same setting (e.g. schools). Third, review inclusion criteria were restricted to prospective evaluations of studies reporting measures of fruit and vegetable intake (or purchasing). Some intervention strategies (e.g. those targeting modification of the food supply) may not be readily amendable to evaluation using conventional prospective designs, or their effects may be more appropriately assessed using other metrics (e.g. sales volume or availability) that can be correlated with population level intakes. Including broader study design and outcome measures in the eligibility criteria of the umbrella review may have identified other relevant reviews. Anecdotally, however, such systematic reviews were not apparent during screening for this review. The included systematic reviews also largely examined intervention strategies that were more commonly implemented in high-income countries, and most of the primary studies included in these systematic reviews were from high-income nations, reflecting the available published literature. Hence, the global applicability of the findings of some strategies may be limited, underscoring the need for both primary studies and reviews specific to intervention strategies undertaken in LMIC.

Notwithstanding its limitations, the review identifies a range of options for health policy makers and practitioners interested in improving the health and wellbeing of communities through strategies to improve fruit and vegetable intake. In particular, the review provides evidence of a beneficial effect for setting-based and fiscal approaches. Many intervention strategies based on the food system (e.g. those targeting agricultural production practices or the supply chain, or more macro-level intervention strategies such as international trade agreements or climate change policies) could have a profound impact on population-level fruit and vegetable consumption; however, their effects have not been reported in systematic reviews. The conduct of primary studies assessing the impact of such approaches and their inclusion in systematic reviews would better support appraisal of their benefits, which in turn would help to strengthen national and international public health nutrition efforts.

## Supplementary Information

**Additional file 1.** Search strategy (contains details of the search terms used in database searches).

**Additional file 2.** Quality assessment of included reviews (contains details on the quality assessments for each review).

**Additional file 3.** Pooled effects of intervention strategies to improve fruit and vegetable uptake.

## Data Availability

The datasets used and/or analysed during the umbrella review are available from the corresponding author on reasonable request.

## Abbreviations

CA: critical appraisal
CI: confidence interval
FAO: Food and Agriculture Organization of the United Nations
GRADE: Grading of Recommendations, Assessment, Development and Evaluations
LMIC: low-and middle-income countries
NCDs: non-communicable diseases
PRIOR: Preferred Reporting Items for Overviews of Reviews
PRISMA: Preferred Reporting Items for Systematic Reviews and Meta-Analyses
RCT: randomised controlled trial
SMD: standardised mean difference
UN: United Nations
US: United States
WCRF: World Cancer Research Fund International
WHO: World Health Organization
