# Supplementary material for: Consolidating evidence on the effectiveness of interventions promoting fruit and vegetable consumption: an umbrella review
Source: Int J Behav Nutr Phys Act. 2021 Jan 11;18:11. doi: 10.1186/s12966-020-01046-y (PMC7798190; doi:10.1186/s12966-020-01046-y)
Supplement: Supplementary file 1 — Additional file 1. Search strategy (contains details of the search terms used in database searches). [file 12966_2020_1046_MOESM1_ESM.docx]

**Additional File 1: Search strategy**

| **Database** | **Vendor** | **Records found** | **Records after de-duplication** |
| --- | --- | --- | --- |
| Academic Search Ultimate | EBSCO | 935 | 418 |
| CINAHL | EBSCO | 318 | 105 |
| Cochrane | Wiley | 36 | 21 |
| Embase | OVID | 2246 | 1020 |
| Medline | OVID | 1870 | 1856 |
| Scopus | Scopus | 1100 | 166 |
| **TOTAL** |  | **6505** | **3586** |

Database(s): **Ovid MEDLINE(R) and Epub Ahead of Print, In-Process & Other Non-Indexed Citations and Daily**1946 to June 09, 2020
Search Strategy:

| **#** | **Searches** | **Results** |
| --- | --- | --- |
| 1 | review.tw. | 1595860 |
| 2 | review/ | 2655682 |
| 3 | (meta-analysis or metaanalysis).tw. | 149570 |
| 4 | meta-analysis/ | 115626 |
| 5 | or/1-4 | 3335364 |
| 6 | exp consumer health information/ or health education, dental/ or health fairs/ or exp health promotion/ or exp patient education as topic/ | 222109 |
| 7 | Primary Prevention/ | 18383 |
| 8 | preventive health services/ or early intervention, educational/ or early medical intervention/ or exp health education/ or exp school health services/ | 274749 |
| 9 | education/ or exp education, nonprofessional/ or education, predental/ or education, premedical/ or exp education, professional/ | 578427 |
| 10 | exp Community Health Services/ | 302186 |
| 11 | exp Preventive Medicine/ | 35254 |
| 12 | prevention.tw. | 536212 |
| 13 | ((Nutrition* or diet* or fruit* or vegetable* or food* or menu*) adj5 (label* or standard* or regulation* or guideline*)).tw. | 38943 |
| 14 | ((Nutrition* or diet* or fruit* or vegetable* or food*) adj5 (tax* or tariff* or subsid* or broadcast* or advertis* or market* or pledge* or self-regulat* or industry reform* or availability or incentive* or procurement* or supply chain* or food system or agriculture*)).tw. | 21808 |
| 15 | 6 or 7 or 8 or 9 or 10 or 11 or 12 or 13 or 14 | 1433149 |
| 16 | exp Fruit/ | 102964 |
| 17 | exp Citrus/ | 9665 |
| 18 | Vegetables/ | 24116 |
| 19 | (fruit* or vegetable* or legume* or citrus*).tw. | 152484 |
| 20 | 16 or 17 or 18 or 19 | 230657 |
| 21 | 5 and 15 and 20 | 3507 |
| 22 | comment/ or editorial/ or letter/ | 1853775 |
| 23 | 21 not 22 | 3491 |
| 24 | animals/ not humans/ | 4672743 |
| 25 | 23 not 24 | 3414 |
| **26** | **limit 25 to yr="2011 -Current"** | **1870** |

Database(s): **Embase**1947 to present
Search Strategy:

| **#** | **Searches** | **Results** |
| --- | --- | --- |
| 1 | review.tw. | 2048888 |
| 2 | "review"/ | 2527411 |
| 3 | (meta-analysis or metaanalysis).tw. | 196742 |
| 4 | meta analysis/ | 188929 |
| 5 | 1 or 2 or 3 or 4 | 3834750 |
| 6 | health education/ or consumer health information/ or patient education/ | 213459 |
| 7 | dental health education/ or school health education/ | 6577 |
| 8 | exp health promotion/ | 98932 |
| 9 | primary prevention/ | 40720 |
| 10 | preventive health service/ | 28748 |
| 11 | exp school health service/ | 22892 |
| 12 | education/ | 438854 |
| 13 | vocational education/ | 11295 |
| 14 | preventive medicine/ | 28116 |
| 15 | prevention.tw. | 723897 |
| 16 | ((Nutrition* or diet* or fruit* or vegetable* or food* or menu*) adj5 (label* or standard* or regulation* or guideline*)).tw. | 53655 |
| 17 | ((Nutrition* or diet* or fruit* or vegetable* or food*) adj5 (tax* or tariff* or subsid* or broadcast* or advertis* or market* or pledge* or self-regulat* or industry reform* or availability or incentive* or procurement* or supply chain* or food system or agriculture*)).tw. | 25435 |
| 18 | 6 or 7 or 8 or 9 or 10 or 11 or 12 or 13 or 14 or 15 or 16 or 17 | 1529322 |
| 19 | exp fruit/ | 142528 |
| 20 | exp Citrus/ | 12337 |
| 21 | exp Citrus/ | 12337 |
| 22 | vegetable/ | 46256 |
| 23 | (fruit* or vegetable* or legume* or citrus*).tw. | 184203 |
| 24 | 19 or 20 or 21 or 22 or 23 | 262394 |
| 25 | 5 and 18 and 24 | 4478 |
| 26 | editorial/ or letter/ or note/ | 2455627 |
| 27 | 25 not 26 | 4463 |
| 28 | limit 27 to human | 3866 |
| **29** | **limit 28 to yr="2011 -Current"** | **2245** |

**CINAHL**

| **#** | **Query** | **Results** |
| --- | --- | --- |
| S1 | AB review | 420,113 |
| S2 | (MH "Systematic Review") OR (MH "Literature Review") OR (MH "Scoping Review") | 103,507 |
| S3 | AB meta-analysis | 51,295 |
| S4 | TI ( meta-analysis or metanaalysis ) OR AB ( meta-analysis or metanaalysis ) | 70,258 |
| S5 | (MH "Meta Analysis") | 50,857 |
| S6 | S1 OR S2 OR S3 OR S4 OR S5 | 498,818 |
| S7 | (MH "Health Education") | 29,194 |
| S8 | (MH "Consumer Health Information+") | 17,106 |
| S9 | (MH "Dental Health Education") | 981 |
| S10 | (MH "Health Fairs") | 548 |
| S11 | (MH "Health Promotion+") | 73,870 |
| S12 | (MH "Patient Education+") | 84,168 |
| S13 | "primary prevention" | 7,116 |
| S14 | "preventive health services" | 12,885 |
| S15 | "early medical intervention" | 6,515 |
| S16 | (MH "Health Education+") | 138,130 |
| S17 | (MH "School Health Services+") | 24,181 |
| S18 | (MH "Education") | 11,985 |
| S19 | (MH "Education, Nonprofessional+") | 154,683 |
| S20 | "predental education" | 9 |
| S21 | (MH "Education, Premedical") | 89 |
| S22 | "professional education" | 3,459 |
| S23 | (MH "Community Health Services+") | 462,434 |
| S24 | "Preventive Medicine" | 13,608 |
| S25 | TI ( ((prevention)) ) OR AB ( ((prevention)) ) | 197,865 |
| S26 | TI ( ((Nutrition* or diet* or fruit* or vegetable* or food* or menu*) n5 (label* or standard* or regulation* or guideline*)) ) OR AB ( ((Nutrition* or diet* or fruit* or vegetable* or food* or menu*) n5 (label* or standard* or regulation* or guideline*)) ) | 14,946 |
| S27 | TI ( ((Nutrition* or diet* or fruit* or vegetable* or food*) n5 (tax* or tariff* or subsid* or broadcast* or advertis* or market* or pledge* or self-regulat* or industry reform* or availability or incentive* or procurement* or supply chain* or food system or agriculture*)) ) OR AB ( ((Nutrition* or diet* or fruit* or vegetable* or food*) n5 (tax* or tariff* or subsid* or broadcast* or advertis* or market* or pledge* or self-regulat* or industry reform* or availability or incentive* or procurement* or supply chain* or food system or agriculture*)) ) | 8,281 |
| S28 | S7 OR S8 OR S9 OR S10 OR S11 OR S12 OR S13 OR S14 OR S15 OR S16 OR S17 OR S18 OR S19 OR S20 OR S21 OR S22 OR S23 OR S24 OR S25 OR S26 OR S27 | 745,470 |
| S29 | (MH "Fruit+") | 28,819 |
| S30 | (MH "Vegetables+") | 27,231 |
| S31 | TI ( (fruit* or vegetable* or legume* or citrus*) ) OR AB ( (fruit* or vegetable* or legume* or citrus*) ) | 30,819 |
| S32 | S29 OR S30 OR S31 | 61,937 |
| **S33** | **S6 AND S28 AND S32 limited 2011+ humans** | **318** |

**COCHRANE**

| **ID** | **Search** | **Hits** |
| --- | --- | --- |
| #1 | MeSH descriptor: [Health Education] explode all trees | 19385 |
| #2 | MeSH descriptor: [Consumer Health Information] explode all trees | 482 |
| #3 | MeSH descriptor: [Health Education, Dental] this term only | 261 |
| #4 | MeSH descriptor: [Health Fairs] this term only | 7 |
| #5 | MeSH descriptor: [Health Promotion] explode all trees | 6272 |
| #6 | MeSH descriptor: [Patient Education as Topic] explode all trees | 8701 |
| #7 | MeSH descriptor: [Primary Prevention] this term only | 840 |
| #8 | MeSH descriptor: [Preventive Health Services] this term only | 484 |
| #9 | MeSH descriptor: [Early Intervention, Educational] this term only | 489 |
| #10 | MeSH descriptor: [Early Medical Intervention] this term only | 372 |
| #11 | MeSH descriptor: [School Health Services] explode all trees | 1540 |
| #12 | MeSH descriptor: [Education] this term only | 574 |
| #13 | MeSH descriptor: [Education, Nonprofessional] explode all trees | 21320 |
| #14 | MeSH descriptor: [Education, Predental] this term only | 0 |
| #15 | MeSH descriptor: [Education, Premedical] this term only | 4 |
| #16 | MeSH descriptor: [Education, Professional] explode all trees | 4769 |
| #17 | MeSH descriptor: [Community Health Services] explode all trees | 13424 |
| #18 | MeSH descriptor: [Preventive Medicine] explode all trees | 190 |
| #19 | prevention:ti,ab | 76657 |
| #20 | ((Nutrition* or diet* or fruit* or vegetable* or food* or menu*) near/5 (label* or standard* or regulation* or guideline*)):ti,ab | 5571 |
| #21 | ((Nutrition* or diet* or fruit* or vegetable* or food*) near/5 (tax* or tariff* or subsid* or broadcast* or advertis* or market* or pledge* or self-regulat* or industry reform* or availability or incentive* or procurement* or supply chain* or food system or agriculture*)):ti,ab | 33612 |
| #22 | {OR #1-#21} | 142737 |
| #23 | MeSH descriptor: [Fruit] explode all trees | 2579 |
| #24 | MeSH descriptor: [Citrus] explode all trees | 446 |
| #25 | MeSH descriptor: [Vegetables] this term only | 1233 |
| #26 | (fruit* or vegetable* or legume* or citrus*):ti,ab | 8326 |
| #27 | {OR #23-#26} | 9985 |
| **#28** | **{AND #22, #27} with Cochrane Library publication date Between Jan 2011 and Jun 2020** | **36** |

**ACADEMIC SEARCH ULTIMATE**

| **#** | **Query** | **Results** |
| --- | --- | --- |
| S1 | TI ( Fruit* or vegetable* or citrus* or legume*) OR AB ( Fruit* or vegetable* or citrus* or legume*) | 212,956 |
| S2 | TI ( “health education” or “consumer health information” or “health fair*” or “health promotion” or “Primary Prevention” or “preventive health service*” or “early medical intervention*” or “school health service*” or education or “Community Health Service*” or “Preventive Medicine” or prevention or ((Nutrition* or diet* or fruit* or vegetable* or food* or menu*) n5 (label* or standard* or regulation* or guideline*)) or ((Nutrition* or diet* or fruit* or vegetable* or food*) n5 (tax* or tariff* or subsid* or broadcast* or advertis* or market* or pledge* or “self-regulat*” or “industry reform*” or availability or incentive* or procurement* or “supply chain*” or “food system” or agriculture*)) ) OR AB ( “health education” or “consumer health information” or “health fair*” or “health promotion” or “Primary Prevention” or “preventive health service*” or “early medical intervention*” or “school health service*” or education or “Community Health Service*” or “Preventive Medicine” or prevention or ((Nutrition* or diet* or fruit* or vegetable* or food* or menu*) n5 (label* or standard* or regulation* or guideline*)) or ((Nutrition* or diet* or fruit* or vegetable* or food*) n5 (tax* or tariff* or subsid* or broadcast* or advertis* or market* or pledge* or “self-regulat*” or “industry reform*” or availability or incentive* or procurement* or “supply chain*” or “food system” or agriculture*)) ) | 1,302,398 |
| S3 | AB ( review or metaanalysis or "meta analysis" ) OR TI ( review or metaanalysis or "meta analysis" ) | 4,031,974 |
| **S4** | **S1 AND S2 AND S3** | **933** |

**SCOPUS**

TITLE-ABS ( fruit* OR vegetable* OR citrus* OR legume* ) AND TITLE-ABS ( "health education" OR "consumer health information" OR "health fair*" OR "health promotion" OR "Primary Prevention" OR "preventive health service*" OR "early medical intervention*" OR "school health service*" OR education OR "Community Health Service*" OR "Preventive Medicine" OR prevention OR ( ( nutrition* OR diet* OR fruit* OR vegetable* OR food* OR menu* ) W/5 ( label* OR standard* OR regulation* OR guideline* ) ) OR ( ( nutrition* OR diet* OR fruit* OR vegetable* OR food* ) W/5 ( tax* OR tariff* OR subsid* OR broadcast* OR advertis* OR market* OR pledge* OR "self-regulat*" OR "industry reform*" OR availability OR incentive* OR procurement* OR "supply chain*" OR "food system" OR agriculture* ) ) ) AND TITLE-ABS ( review OR metaanalysis OR "meta analysis" ) AND ( LIMIT-TO ( PUBYEAR , 2020 ) OR LIMIT-TO ( PUBYEAR , 2019 ) OR LIMIT-TO ( PUBYEAR , 2018 ) OR LIMIT-TO ( PUBYEAR , 2017 ) OR LIMIT-TO ( PUBYEAR , 2016 ) OR LIMIT-TO ( PUBYEAR , 2015 ) OR LIMIT-TO ( PUBYEAR , 2014 ) OR LIMIT-TO ( PUBYEAR , 2013 ) OR LIMIT-TO ( PUBYEAR , 2012 ) OR LIMIT-TO ( PUBYEAR , 2011 ) ) AND ( EXCLUDE ( DOCTYPE , "no" ) OR EXCLUDE ( DOCTYPE , "ed" ) OR EXCLUDE ( DOCTYPE , "er" ) ) AND ( LIMIT-TO ( EXACTKEYWORD , "Human" ) OR LIMIT-TO ( EXACTKEYWORD , "Humans" ) )

**Targeted Google Scholar**

The following search terms were entered individually into Google Scholar with the first 30 citation examined.

‘Systematic review of food labelling to improve fruit and vegetable intake’

‘Systematic review of food standards to improve fruit and vegetable intake’

‘Systematic review of food regulations to improve fruit and vegetable intake’

‘Systematic review of food guidelines to improve fruit and vegetable intake’

‘Systematic review of food tax to improve fruit and vegetable intake’

‘Systematic review of tariff to improve fruit and vegetable intake’

‘Systematic review subsidies to improve fruit and vegetable intake’

‘Systematic review of advertising to improve fruit and vegetable intake’

‘Systematic review of marketing to improve fruit and vegetable intake’

‘Systematic review of incentives to improve fruit and vegetable intake’

‘Systematic review of procurement strategies to improve fruit and vegetable intake’

‘Systematic review of supply chain interventions to improve fruit and vegetable intake’

‘Systematic review of food system interventions to improve fruit and vegetable intake’

‘Systematic review of agricultural interventions to improve fruit and vegetable intake’

**Google search for grey literature**

The following phrase was searched. A total of 20 citations came back which were all screened for inclusion. No additional reviews were identified.

‘systematic review of fruit and vegetable intervenitons’
