# Supplementary material for: Consolidating evidence on the effectiveness of interventions promoting fruit and vegetable consumption: an umbrella review
Source: Int J Behav Nutr Phys Act. 2021 Jan 11;18:11. doi: 10.1186/s12966-020-01046-y (PMC7798190; doi:10.1186/s12966-020-01046-y)
Supplement: Supplementary file 2 — Additional file 2. Quality assessment of included reviews (contains details on the quality assessments for each review). [file 12966_2020_1046_MOESM2_ESM.docx]

**Additional File 2.** Quality assessment of included reviews

| **Author, year of publication** | **CA1** | **CA2** | **CA3** | **CA4** | **CA5** | **CA6** | **CA7** | **CA8** | **CA9** | **CA10** | **CA11** | **Overall quality** |
| --- | --- | --- | --- | --- | --- | --- | --- | --- | --- | --- | --- | --- |
| Afshin et al., 2015(45) | Yes | Yes | Unsure | Unsure | No | NA | No | Yes | No | Yes | Yes | Medium |
| Afshin et al., 2017(46) | Yes | Yes | Yes | No | Yes | Yes | Yes | Yes | Yes | Yes | Yes | High |
| Carter et al., 2018(41) | Yes | Yes | Yes | No | Yes | Yes | Unsure | Yes | No | NA | Yes | High |
| Champion et al., 2019(38) | Yes | Yes | Yes | Yes | Yes | Yes | Yes | Yes | Yes | NA | Yes | High |
| Cornelsen et al., 2015(49) | Yes | Yes | No | Yes | No | NA | No | Yes | No | Yes | Yes | Medium |
| DeCosta et al., 2017(18) | No | Yes | Unsure | No | No | NA | Unsure | Yes | No | Yes | Yes | Medium |
| Feltner et al., 2016(48) | No | Yes | Yes | Yes | Yes | Yes | Yes | Yes | No | NA | Yes | High |
| Girard et al., 2012(37) | No | Yes | No | Yes | No | NA | Unsure | Yes | No | Yes | Yes | Medium |
| Hendren et al., 2017(50) | Yes | Yes | Yes | No | Yes | Yes | Yes | Yes | No | NA | Yes | High |
| Hendrie et al., 2017(42) | Yes | Yes | Yes | No | Yes | Unsure | No | Yes | No | Yes | Yes | Medium |
| Hodder et al., 2020(19) | Yes | Yes | Yes | Yes | Yes | Yes | Yes | Yes | Yes | Yes | Yes | High |
| Hollis-Hansen et al., 2019(35) | Yes | Yes | Yes | No | No | NA | Unsure | Yes | No | Yes | Yes | Medium |
| Hsiao et al., 2018(36) | Yes | Yes | Yes | No | Yes | Yes | No | Yes | No | Yes | Yes | High |
| Langford et al., 2014(39) | Yes | Yes | Yes | Yes | Yes | Yes | Yes | Yes | Yes | Yes | Yes | High |
| Micha et al., 2018(43) | Yes | Yes | Yes | Yes | Yes | Yes | Yes | Yes | Yes | Yes | Yes | High |
| Patnode et al., 2017(44) | Yes | Yes | Yes | Yes | Yes | Yes | Yes | Yes | Yes | NA | Yes | High |
| Rochira et al., 2020(47) | Yes | Yes | No | Yes | Yes | Unsure | Yes | Yes | Yes | Yes | Yes | High |
| Rodriguez et al., 2019(22) | Yes | Yes | Yes | Yes | Yes | Unsure | Unsure | Yes | Yes | NA | Yes | High |
| Silveira et al., 2011(40) | Yes | Yes | Yes | No | Yes | Yes | No | Yes | No | Yes | No | Medium |

Critical appraisal (CA) 1: Is the review question clearly and explicitly stated? CA2: Were the inclusion criteria appropriate for the review question? CA3: Was the search strategy appropriate? CA4: Were the sources and resources used to search for studies adequate? CA5: Were the criteria for appraising studies appropriate? CA6: Was critical appraisal conducted by two or more reviewers independently? CA7:Were there methods to minimise errors in data extraction? CA8: Were the methods used to combine studies appropriate? CA9: Was the likelihood of publication bias assessed? CA10: Were recommendations for policy and/or practice supported by the reported data? CA11: Were the specific directives for new research appropriate? NA = Not Applicable. Overall quality appraisal: reviews as low quality where 33% or less of the criteria are met, medium quality where 34–66% of criteria are met, and high quality where 67% or more of criteria are met.

**Additional information on scoring critical appraisal criteria**

Where studies clearly provided evidence of meeting the critical appraisal (CA) criteria, these were scored yes. If studies clearly did not meet the criteria they were scored no. For example, CA4 asks ‘Were the sources and resources used to search for studies adequate?’. To score a ‘yes’ included reviews needed to search more than two databases, provided evidence of subject heading, indexing and keywords, and explained limits on searches or they were intuitive. A ‘no’ was given to a review that clearly did not achieve this (e.g. did not search more than two databases). ‘Unsure’ was scored for studies which did not provide enough information to determine a yes/no scoring clearly. ‘Not applicable’ was used if the critical appraisal criteria was not relevant to the review, e.g. “CA6: Was critical appraisal conducted by two or more reviewers independently” was scored not applicable for some reviews if the previous question ‘CA5: Were the criteria for appraising studies appropriate?’ was scored no. This is because the included studies did not quality appraise their included studies, and therefore the question about critically appraising in duplication is irrelevant.
