# Supplementary material for: Consolidating evidence on the effectiveness of interventions promoting fruit and vegetable consumption: an umbrella review
Source: Int J Behav Nutr Phys Act. 2021 Jan 11;18:11. doi: 10.1186/s12966-020-01046-y (PMC7798190; doi:10.1186/s12966-020-01046-y)
Supplement: Supplementary file 3 — Additional file 3. Pooled effects of intervention strategies to improve fruit and vegetable uptake. [file 12966_2020_1046_MOESM3_ESM.docx]

**Additional File 3**. Pooled effects of interventions to improve fruit and vegetable intake


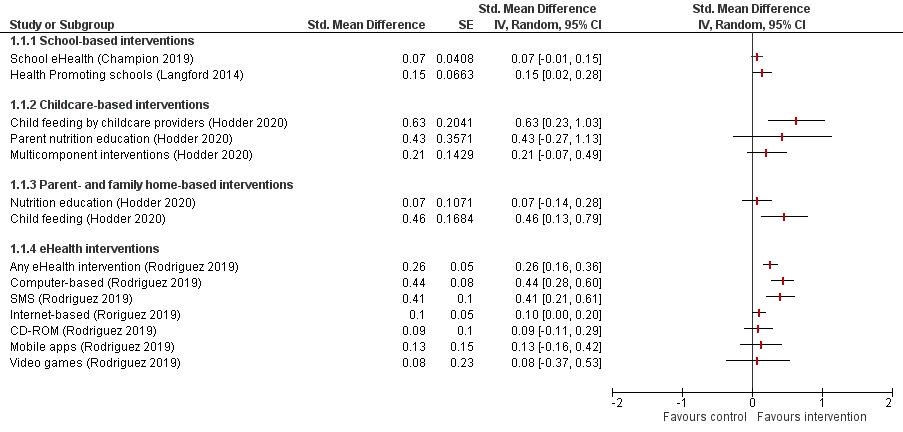


Note: The results presented in this plot are taken from separate systematic reviews that differ from each other in a number of ways and should not be used to draw conclusions about the relative effects of interventions assessed across different reviews
